# Postoperative Sinus Tract: A Rare Occurrence Following Laparoscopic Cholecystectomy

**DOI:** 10.7759/cureus.71328

**Published:** 2024-10-12

**Authors:** Vinayak V Kshirsagar, Rushi Kanani, Prashanth I AM, Hima Shree

**Affiliations:** 1 Department of General Surgery, Dr. D.Y. Patil Medical College, Hospital and Research Centre, Dr. D.Y. Patil Vidyapeeth (Deemed to be University), Pune, IND

**Keywords:** abdominal surgery, chronic inflammation, granulation tissue, laparoscopic cholecystectomy, postoperative complication, sinus tract

## Abstract

A sinus tract is a rare clinical condition characterized by a blind-ended tubular structure extending from deeper tissues to the skin, commonly occurring postoperatively, especially after gastrointestinal surgeries. Its pathogenesis often involves the breakdown of fatty tissue, pyogenic infections, or the retention of foreign bodies such as surgical implants or sutures. One notable scenario includes sinus tract formation following laparoscopic cholecystectomy due to retained gallstones. Chronic cases are often associated with inadequate drainage of inflamed tissues, leading to granulation and subsequent scar tissue formation. In our case, a 34-year-old male presented with persistent discharge from the infraumbilical region for three months, accompanied by intermittent low-grade fever over the preceding two days and continuous lower abdominal pain. The patient had no significant comorbidities or harmful habits but had undergone a laparoscopic cholecystectomy six years earlier. Ultrasonography (USG) revealed an area of subcutaneous fat liquefaction in the left lumbar region, along with an extraperitoneal fistulous tract draining into the suprapubic region. The patient was treated surgically through sinus tract excision.

## Introduction

The sinus tract, a blind-ended tubular structure extending from deeper tissue layers to the outermost layer of skin, represents a rare nosological entity, with a higher incidence in patients who underwent gastrointestinal operations, particularly in cases of infected surgeries, traumas, and damage control procedures [1,2]. It has been postulated that the pathogenesis may be either the disintegration of fatty tissue, pyogenic infection, or the retention of foreign materials, such as implants and surgical threads [1-3]. The latter is also suspected in sinuses occurring after laparoscopic cholecystectomy, where one of the gall stones is retained.

Laparoscopic cholecystectomy has become the "gold standard" for treating symptomatic gallstone disease, offering several advantages over open cholecystectomy. These include reduced pain, faster recovery, improved cosmetic results, and a lower risk of incisional hernia. However, the laparoscopic technique is not without complications, and surgeons are encountering new challenges that are uncommon with the traditional open method. These include traumatic injuries caused by the Veress needle, trocar, or laparoscopic instruments, as well as thermal injuries from diathermy due to accidental coupling or unintended contact with internal organs. Port-related complications, such as infections, also require careful management and training, particularly for less experienced surgeons.

There are two types of port site infections (PSIs). The first type occurs within a week of surgery and is typically caused by Gram-positive or Gram-negative bacteria. This infection generally resolves with appropriate wound care and antibiotics within a few days. The second type, chronic port site infection, emerges three to four weeks after surgery once wound healing has taken place. This is usually due to atypical mycobacteria, a group of mycobacteria distinct from the Mycobacterium tuberculosis complex, which have an incubation period of three to four weeks and do not respond to standard antibiotics [4].

Chronic PSI progress through five clinical stages as follows [5]. (i) First stage: A tender nodule develops near the port site, typically appearing around four weeks after surgery. (ii) Second stage: The nodule increases in size, with heightened tenderness and signs of inflammation, eventually leading to the formation of an abscess. (iii) Third stage: Pain decreases as the abscess discharges purulent material, accompanied by necrosis of the surrounding skin. (iv) Fourth stage: A chronic sinus forms, discharging white or serous fluid. (v) Fifth stage: Hyperpigmentation develops around the sinus, along with the appearance of multiple nodules at different locations.

The risk of the illness becoming chronic significantly increases due to inadequate tissue drainage, a result of the inflammatory process occurring at the surgical incision site. The culmination of this degenerative progression is the gradual creation of granulation tissue, followed by scar tissue formation. Creating an infected sinus in the abdominal wall, described as a sinus in clinical terms [6-8], is the end outcome of this gradual process. To develop effective treatments, it is essential to have a thorough understanding of the complex chain of events that guides the formation of the sinuses. Without this understanding, it is possible to experience chronic recurrences and significant clinical problems. Hence, we present this rare occurrence of sinus tract as follows.

## Case presentation

A 34-year-old male arrived at the surgical department with a three-month history of discharge from the infraumbilical region, along with intermittent low-grade fever for the past two days and lower abdominal pain.

The patient had no comorbidities or habits detrimental to health. However, he reported having undergone a laparoscopic cholecystectomy six years prior. Upon further inquiry, the patient revealed a history of multiple episodes of discharge from the port site, which had been managed conservatively with oral antibiotics and anti-inflammatory medications.

**Figure 1:**
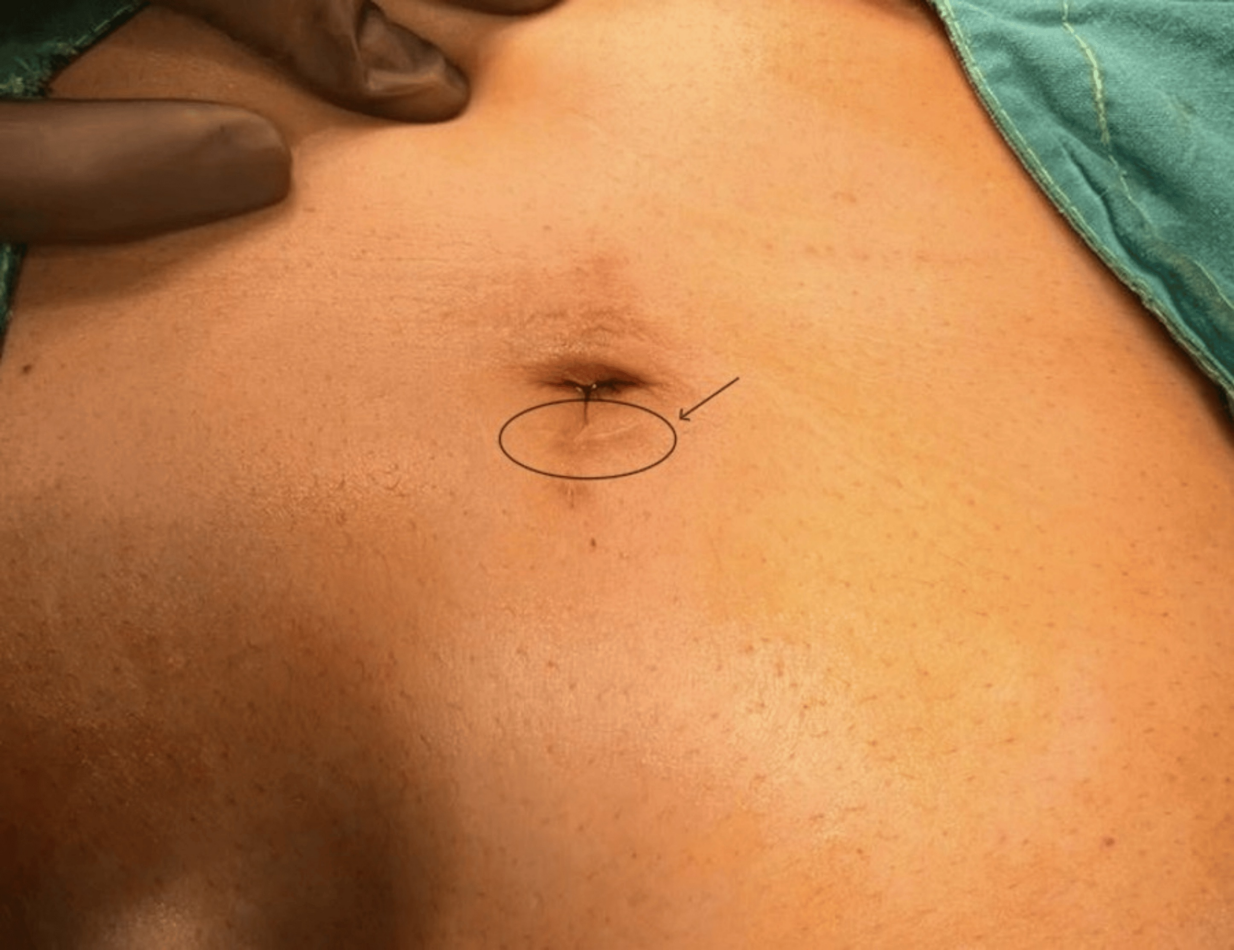
Discharging sinus from port site scar

On clinical examination, the patient was afebrile with stable hemodynamics. The abdominal examination was generally unremarkable, except for an actively discharging sinus from the scar of the previous laparoscopic port (Figure 1). The discharge was serosanguinous, minimal, and non-foul-smelling. Palpation revealed surrounding induration and a firm, mobile mass measuring 2 cm × 3 cm beneath the umbilicus. The rest of the systemic examination was normal.

An abdominal and pelvis ultrasonography (USG) revealed an area of subcutaneous fat liquefaction in the left lumbar region and an extraperitoneal fistulous tract extending to the suprapubic area, measuring 3.3 cm × 4.6 cm. A fibroma was also noted on the left side of the rectus abdominis muscle.

The patient underwent surgery for exploration and excision of the sinus tract.

**Figure 2:**
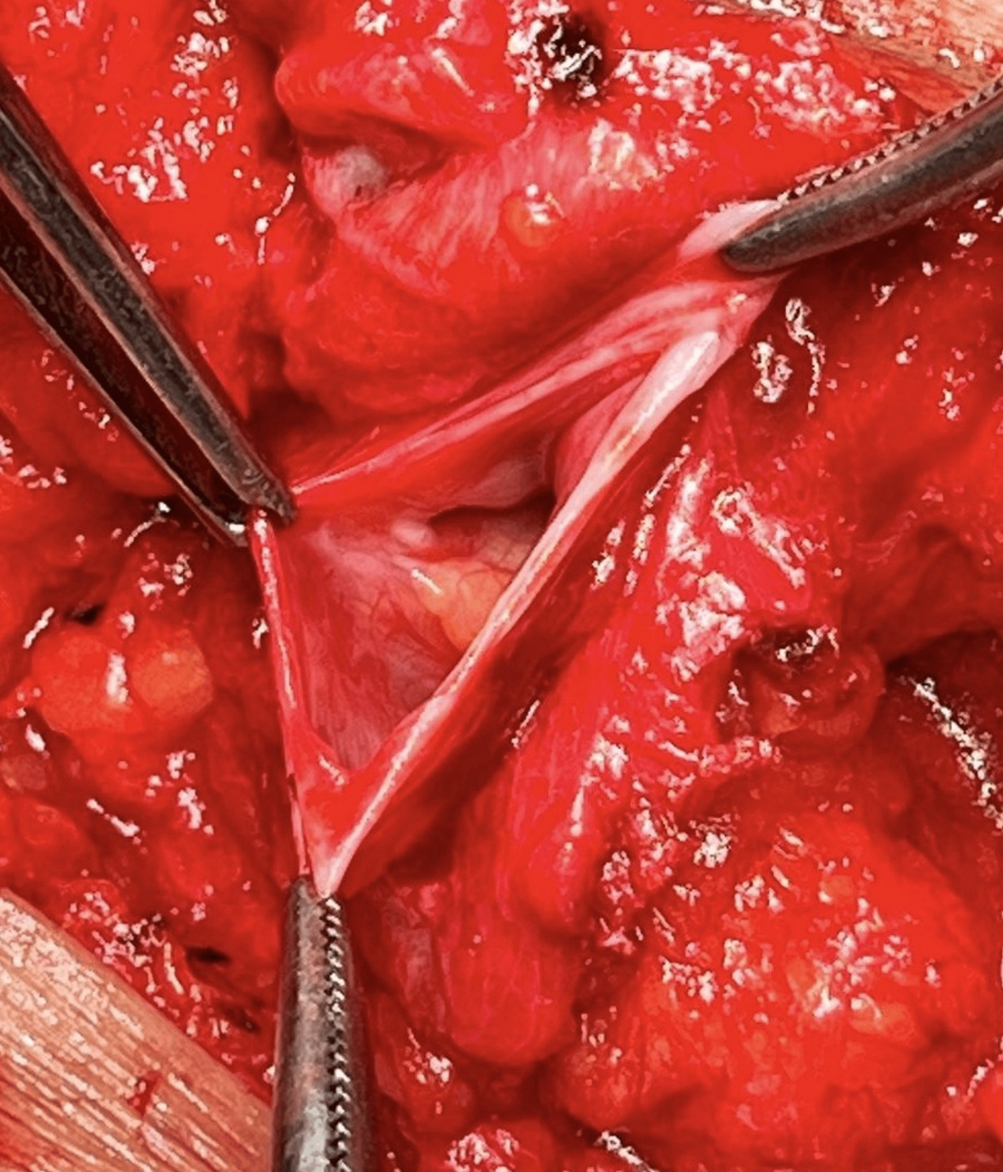
Sinus tract extending to abdominal cavity

**Figure 3:**
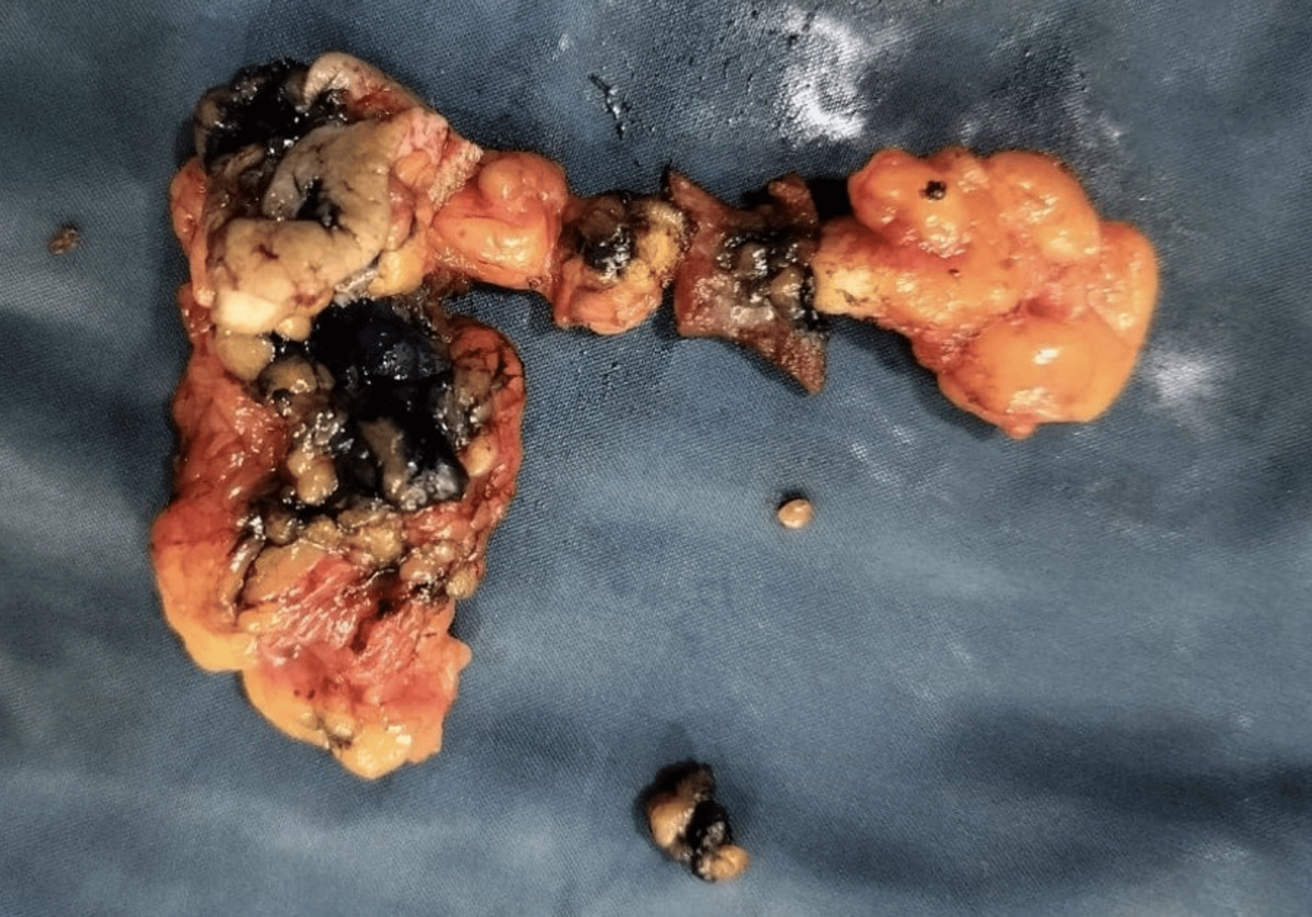
Excised specimen of sinus tract

**Figure 4:**
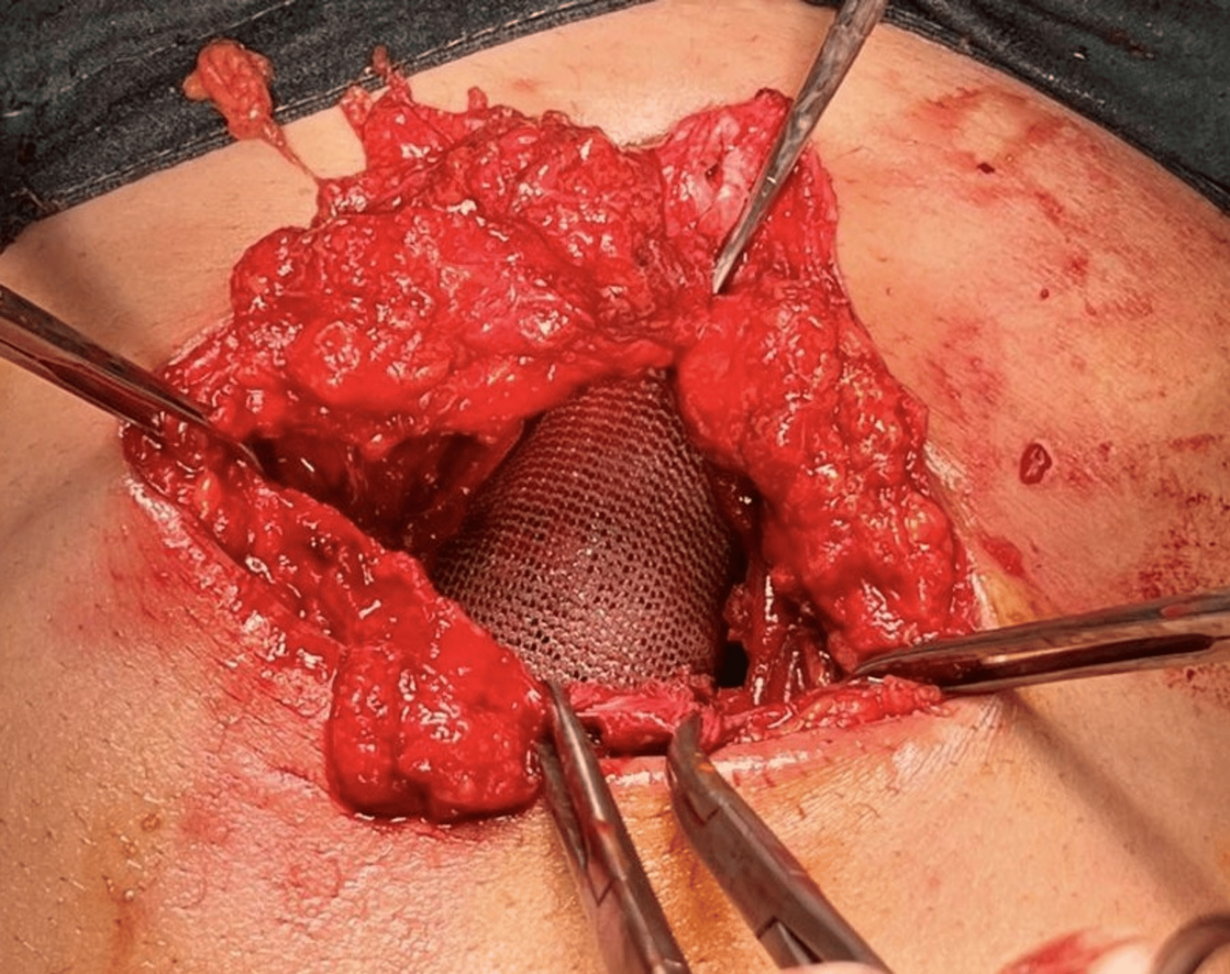
Prolene mesh fixation

Intraoperatively, a sinus tract extending into the abdominal cavity was identified (Figure 2). The sinus tract was circumferentially dissected and opened. There was evidence of fibrosis and necrosis of the subcutaneous tissue, which was entirely excised (Figure 3). No gallstone fragments were found, nor was there any active source of infection. After a thorough saline wash, the communication with the intra-abdominal cavity was obliterated and reinforced with a Prolene mesh (Figure 4).

Histopathology confirmed a sinus tract with chronic inflammation, with no evidence of granuloma or malignancy. The patient is now on a regular follow-up schedule every six months, with no signs of discharge or recurrence.

## Discussion

An intra-abdominal sinus tract, like those in other locations, is marked by ongoing discomfort, skin redness, swelling, and the discharge of serous or purulent fluid [9,10]. The sinus tract has a chronic nature, which these symptoms confirm. Laboratory tests are significant in determining whether or not an infectious illness is present. The most typical findings of these tests are leukocytosis and high inflammatory markers [9]. Clinical suspicion becomes relevant in individuals who continue to experience leakage, even while receiving antibiotic treatment and having percutaneous drainage performed. Nevertheless, it is of the utmost importance to consider other possible diagnoses, such as tuberculosis and cancer treatments [11].

Infections caused by atypical mycobacteria are more commonly seen after laparoscopic procedures and are rare following open surgeries. This is primarily because instruments used in open surgeries are typically sterilized using an autoclave. In contrast, laparoscopic instruments have an insulating layer that limits the use of autoclaving for sterilization [12]. These instruments are often cleaned with tap water, which can be a good source of atypical mycobacteria, and are then disinfected using 2.0-2.5% glutaraldehyde for at least 20 minutes [13]. In our case also, instruments were washed with tap water and sterilized using a CIDEX solution, which can be the reason for this tract formation.

If these instruments are not thoroughly cleaned before being placed in the glutaraldehyde or CIDEX solution, blood and charred tissue residues may remain. The endospores on the contaminated instruments can then be introduced into the subcutaneous tissue, where they germinate over the course of three to four weeks, eventually leading to the appearance of clinical signs and symptoms [4].

One of the most critical factors in avoiding difficulties during surgical procedures is the type of thread that is used. It has been demonstrated that the danger of adhesion or abscess development is reduced when using monofilament or absorbable threads [14]. A comprehensive systematic review of 15 studies involving a large sample of 5,875 patients concluded that polyglactin threads are superior to PDS, polypropylene, and nylon threads [15]. This review highlighted the decreased fistula or sinus formation incidence when polyglactin threads are employed.

The difficulty in clinically assessing a fistula or sinus's size, depth, and involved structures underscores the importance of imaging examinations for accurate diagnosis and optimal surgical planning. Magnetic resonance imaging (MRI) and computed tomography (CT), enhanced by three-dimensional reconstruction, offer precise details about the lesions and their relationships with surrounding structures. In contrast, USG excels in real-time visualization, which is particularly beneficial for examining superficial lesions [2,15]. The restriction of contrasted imaging in terms of visualizing the anatomical relationships of nearby tissues is something that must be taken into consideration. However, it is effective in tracing the course.

Draining the infected material from the sinus passage and resecting the tract are the two main components of the treatment for sinusitis. Although endoscopic therapies are being studied to reduce treatment costs and morbidity, clear evidence regarding their effectiveness remains lacking [16]. The decision on whether or not antibiotic therapy is required should be made individually, considering various aspects, including the patient's clinical response, bacterial resistance, and positive microbiological culture results. A multidisciplinary approach involving surgeons, infectious disease specialists, and radiologists is essential for delivering comprehensive and effective treatment.

## Conclusions

A sinus tract that communicates with an intra-abdominal cavity is a rare but serious complication that can arise following laparoscopic cholecystectomy. Chronic port site infections are a common challenge for laparoscopic surgeons in developing countries. When a wound infection arises three to four weeks or more after surgery, accompanied by poor antibiotic response and ongoing pus discharge from the port site, atypical mycobacterial infection should be suspected. This indolent yet progressive condition, if not promptly addressed, poses significant risks, including the potential spread of infection and, in severe cases, malignant transformation. Adhering to proper sterilization methods and providing effective treatment are crucial for significantly reducing morbidity.
